# The Distribution and Host Shifts of Cotton-Melon Aphids in Northern China

**DOI:** 10.1371/journal.pone.0152103

**Published:** 2016-03-22

**Authors:** Jun-Yu Luo, Shuai Zhang, Li Wang, Li-Min Lv, Chun-Yi Wang, Chun-Hua Li, Xiang-Zhen Zhu, Zhi-Guo Zhou, Jin-Jie Cui

**Affiliations:** 1 Key Laboratory of Crop Physiology & Ecology, Ministry of Agriculture, Nanjing Agricultural University, Nanjing, 210095, Jiangsu, China; 2 State Key Laboratory of Cotton Biology, Institute of Cotton Research, Chinese Academy of Agricultural Sciences, Anyang, 455000, Henan, China; National Key Laboratory of Crop Genetic Improvement, CHINA

## Abstract

*Aphis gossypii* Glover (Hemiptera: Aphididae) is a serious pest of cotton in northern China. A microsatellite analysis was used to characterize the genetic structure of *A*. *gossypii* populations from different geographic, host plant, and seasonal populations in 2014. Among 906 individuals, 507 multilocus genotypes were identified, with genotypic richness values of 0.07–1.00 for the populations. We observed moderate levels of genetic differentiation among geographic populations (F_ST_ = 0.103; 95% confidence interval: 0.065–0.145) and host plant populations (F_ST_ = 0.237; 95% confidence interval: 0.187–0.296). A Mantel test of isolation by distance revealed no significant correlations between Slatkin’s linearized F_ST_ and the natural logarithm of geographic distance. A Bayesian analysis of population genetic structures identified three clusters. An analysis of molecular variance revealed significant differences among the three clusters (*F* = 0.26596, *P* < 0.0001), among seasons (*F* = 0.04244, *P* = 0.00381), and among host populations (*F* = 0.12975, *P* = 0.0029). Thus, the *A*. *gossypii* populations in northern China exhibit considerable genotypic diversity. Additionally, our findings indicated that the 31 analyzed populations could be classified as one of three host biotypes (i.e., cotton, cucumber, and pomegranate biotypes). There were also clear seasonal effects on population genetic structure diversity among aphids collected from Anyang.

## Introduction

The cotton-melon aphid, *Aphis gossypii* Glover, is a polyphagous species with a worldwide distribution. Economic losses caused by this aphid result from its feeding on agricultural crops, the production of honeydew secretions on lint (i.e., sticky cotton), and the fact it serves as a vector for viral diseases [1, 2]. This pest colonizes more than 600 plant species, including many important crops, such as cotton, cucurbits, citrus, aubergine, potato, and okra [1]. *Aphis gossypii* is an important pest of cotton in northern China, causing yield losses during the seedling stage [3].

*Aphis gossypii* has a highly variable life cycle. The aphid is considered to be anholocyclic in most places where it is found, including in Europe and Africa, where it reproduces continuously by apomictic parthenogenesis [1, 4]. However, this aphid can be holocyclic in regions with very harsh winters, such as Japan, China, Korea, India, and the United States of America, where it reproduces sexually, with a few woody plants serving as primary hosts. More than 10 of these primary hosts are extremely abundant in China, including *Hibiscus syriacus*, *Zanthoxylum simulans*, and *Punica granatum* [5–7]. In China, *A*. *gossypii* eggs hatch in March, and two to three generations are produced on primary hosts before alate adults develop because of overcrowding and a diminishing food supply. Alate adults migrate to cotton fields, where seedlings emerge in late April to mid-May. In autumn, these morphs return to the primary hosts, where the gynoparae produce oviparae, which mate and produce eggs [5].

The genetic structure of aphid populations is associated with the spatial patterns of selection pressures resulting from biotic and abiotic factors, such as climate, habitat distribution, dispersal abilities, insecticides, and life cycle [8–10]. Aphid biotypes are defined based on the ability to feed on specific hosts within the host range of the species [7]. Genetic diversity is correlated with host type, with host-specific *A*. *gossypii* biotypes detected in many countries and regions [11, 12]. The existence of *A*. *gossypii* biotypes was determined by host transference experiments [13]. Polymorphisms in microsatellite or simple sequence repeat (i.e., tandem repeats of simple nucleotide sequences) loci have been used to analyze the population genetic structures of many organisms [14]. Eight *A*. *gossypii* microsatellite loci were described in 1999 [15], and have since been widely used in this species [10, 13, 14, 16–19].

In this study, eight microsatellite markers were used to characterize the population structure of *A*. *gossypii* aphids collected from cotton plants at 20 locations in the North China Plain in Henan, Hebei, and Shandong provinces. We also compared the genetic structures of *A*. *gossypii* populations from three host plants in mid-May and late August in Anyang, Henan Province.

## Results

### Sample identification

The polymerase chain reaction (PCR) and sequencing results revealed that all samples collected in cotton fields were *A*. *gossypii*. Some samples collected on hibiscus, pomegranate, and Chinese prickly ash contained other species of aphids, with *A*. *gossypii* comprising 30–95% of all aphids [20].

### Genetic diversity

The microsatellite locus Ago84 failed to amplify in 44.3% of individuals. Therefore, samples were genotyped at seven microsatellite loci. Among the 906 individuals, 507 multilocus genotypes (MLGs) were distinguished, with each population having 3–42 MLGs. The ratio of MLGs to individuals sampled (M/I) in a population was 0.097–1.000. In the populations SDWC, HBWX, HBQZ, HBLZ, SDSX, HNWS, HNSQ, HNLY, and HNYL, the M/I ratios were less than 0.500, and the lowest ratio was observed for HNSQ (0.097) (Table 1). The allelic richness values were 1.45–3.23, with an average of 2.34. The heterozygosity deficit indicates the level of inbreeding, and we determined that six of seven loci exhibited heterozygote deficits. These deficits were observed in three of five Hebei populations, three of five Henan populations, and two of nine Shandong populations. Approximately half of the geographic populations met the Hardy-Weinberg equilibrium (HWE) criteria for panmictic sexual reproduction, while the other half underwent inbreeding. Heterozygote deficits were also observed in half of the Anyang populations, suggesting the prevalence of inbreeding in these populations. The observed proportion of heterozygotes (H_O_) and the expected proportion of heterozygotes (H_E_) were 0.21–0.60 and 0.22–0.62, respectively. The H_O_ value was lower than expected in 20 populations. After correcting the dataset for null alleles using the EM algorithm, the H_O_ and H_E_ values increased to 0.24–0.70 and 0.22–0.64, respectively (Table 1).

**Table 1:** Genetic diversity measures estimated using six microsatellites in 34 *Aphis gossypii* populations.

PN	Collection site (City, Province)	Host plant	AP	CD	CL (GPS)	IND	MLG	M/I ratio	A	AR	Ho^R^/Ho^C^	H_E_^R^/H_E_^C^	F_IS_
1	Nanpi, HB	Cotton	HBNP	Aug, 2014	37°58'53.1N 116°49'34.1E	29	22	0.76	4.43	2.16	0.43/0.58	0.51/0.57	0.16*
2	Laoling, SD	Cotton	SDLL	Aug, 2014	37°37'18.8N 117°09'13.5E	20	12	0.60	4.29	2.72	0.44/0.61	0.51/0.56	0.15*
3	Zhaoqiang, HB	Cotton	HBZQ	Aug, 2014	37°31'25.4N 115°39'22.5E	32	18	0.56	4.29	2.70	0.42/0.57	0.52/0.57	0.21*
4	Wucheng, SD	Cotton	SDWC	Aug, 2014	37°11'35.2N 116°05'42.1E	33	12	0.36	3.57	2.32	0.48/0.53	0.45/0.47	-0.01
5	Xiajing, SD	Cotton	SDXJ	Aug, 2014	37°00'43.2N 116°00'56.3E	13	8	0.62	3.29	2.42	0.52/0.52	0.44/0.44	-0.19
6	Weixian,HB	Cotton	HBWX	Aug, 2014	36°58'59.6N 115°17'37.7E	34	11	0.32	3.14	2.25	0.44/0.52	0.46/0.48	0.05
7	Linqing, SD	Cotton	SDLQ	Aug, 2014	36°53'51.5N 115°49'54.4E	31	23	0.74	4.29	2.30	0.55/0.66	0.49/0.52	-0.10
8	Quzhou, HB	Cotton	HBQZ	Aug, 2014	36°47'02.6N 115°00'06.3E	31	7	0.23	2.71	1.89	0.33/0.33	0.35/0.35	0.11*
9	Qiuxian, HB	Cotton	HBQX	Aug, 2014	36°47'02.6N 115°00'06.3E	14	11	0.79	3.57	2.23	0.56/0.62	0.5/0.52	-0.12
10	Linzhang, HB	Cotton	HBLZ	Aug, 2014	36°20'11.3N 114°33'47.4E	29	3	0.10	1.57	1.48	0.24/0.24	0.22/0.22	-0.03
11	Chengwu, SD	Cotton	SDCW	Aug, 2014	35°04'23.7N 116°07'49.7E	25	15	0.60	3.71	2.62	0.52/0.63	0.55/0.56	-0.01
12	Shanxian, SD	Cotton	SDSX	Aug, 2014	34°47'58.7N 115°59'57.6E	48	5	0.10	2.86	2.26	0.57/0.57	0.47/0.47	-0.22
13	Jingxiang, SD	Cotton	SDJX	Aug, 2014	35°01'18.7N 116°18'22.5E	8	6	0.75	3.43	2.61	0.4/0.6	0.58/0.61	0.28*
14	Caoxian, SD	Cotton	SDCX	Aug, 2014	34°48'21.6N 115°49'27.6E	14	7	0.50	2.71	1.93	0.47/0.57	0.47/0.49	-0.05
15	Weishi, HN	Cotton	HNWS	Aug, 2014	34°03'42.6N 114°27'10.9E	46	15	0.33	3.71	2.34	0.34/0.54	0.5/0.54	0.45*
16	Shangqiu, HN	Cotton	HNSQ	Aug, 2014	34°31'54.9N 115°42'26.8E	31	3	0.10	2.14	1.76	0.48/0.48	0.35/0.35	-0.33
17	Luyi, HN	Cotton	HNLY	Aug, 2014	33°50'33.1N 115°32'41.1E	38	15	0.39	4.29	2.43	0.51/0.62	0.54/0.57	0.06*
18	Yanling, HN	Cotton	HNYL	Aug, 2014	33°50'30.5N 114°14'11.4E	24	6	0.25	3.14	2.45	0.45/0.45	0.46/0.46	0.04
19	Xihua, HN	Cotton	HNXH	Aug, 2014	33°45'58.6N 114°14'14.1E	28	15	0.54	4.29	2.49	0.47/0.57	0.54/0.56	0.13*
20	Anyang, HN	Hibiscus	HI^1^-May	May, 2014	36°5'34.8"N 114°31'47.19"E	60	42	0.70	5.14	2.74	0.6/0.7	0.59/0.62	-0.03
21	Anyang, HN	Pomegranate	PO^1^-May	May, 2014	36°5'34.8"N 114°31'47.19"E	41	25	0.61	3.14	1.59	0.21/0.28	0.28/0.29	0.16*
22	Anyang, HN	Chinese prickly ash	CH^1^-May	May, 2014	36°5'34.8"N 114°31'47.19"E	48	33	0.69	5.00	2.64	0.51/0.51	0.53/0.53	0.07
23	Anyang, HN	Cotton	COW^2^-May	May, 2014	36°5'34.8"N 114°31'47.19"E	13	12	0.92	4.43	3.11	0.58/0.64	0.62/0.62	0.05*
24	Anyang, HN	Cucumber	CUW^2^-May	May, 2014	36°5'34.8"N 114°31'47.19"E	11	7	0.64	1.86	1.45	0.24/0.24	0.23/0.23	-0.09
25	Anyang, HN	Zucchini	ZUW^2^-May	May, 2014	36°5'34.8"N 114°31'47.19"E	36	26	0.72	5.00	2.37	0.39/0.49	0.52/0.56	0.27*
26	Anyang, HN	Muskmelon	MUW^2^-May	May, 2014	36°5'34.8"N 114°31'47.19"E	13	10	0.77	2.57	1.56	0.32/0.32	0.3/0.3	-0.08
27	Anyang, HN	Muskmelon	MU^1^-May	May, 2014	36°5'34.8"N 114°31'47.19"E	18	10	0.56	2.57	1.83	0.31/0.31	0.28/0.28	-0.12
28	Anyang, HN	Kidney bean	KIW^2^-May	May, 2014	36°5'34.8"N 114°31'47.19"E	5	4	0.80	2.71	2.16	0.46/0.5	0.46/0.48	-0.04
29	Anyang, HN	Cucumber	CUW^2^-Aug	Aug, 2014	36°5'34.8"N 114°31'47.19"E	6	5	0.83	3.00	1.86	0.36/0.36	0.41/0.41	0.14*
30	Anyang, HN	Cotton	COW^2^-Aug	Aug, 2014	36°5'34.8"N 114°31'47.19"E	7	7	1.00	4.00	2.92	0.55/0.55	0.59/0.59	0.07
31	Anyang, HN	Zucchini	ZUW^2^-Aug	Aug, 2014	36°5'34.8"N 114°31'47.19"E	14	13	0.93	4.00	2.95	0.43/0.56	0.61/0.63	0.28*
32	Anyang, HN	Cotton	COW^2^-Sep	Sep, 2014	36°5'34.8"N 114°31'47.19"E	36	36	1.00	5.14	2.44	0.52/0.59	0.57/0.58	0.08*
33	Anyang, HN	Cucumber	CUW^2^-Sep	Sep, 2014	36°5'34.8"N 114°31'47.19"E	31	25	0.81	5.57	3.18	0.43/0.58	0.62/0.64	0.28*
34	Anyang, HN	Zucchini	ZUW^2^-Sep	Sep, 2014	36°5'34.8"N 114°31'47.19"E	39	38	0.97	5.71	3.23	0.46/0.52	0.62/0.64	0.23*

*heterozygote deficiency (*P* < 0.05) among the populations HBNP, SDLL, HBZQ, HBQZ, SDJX, HNWS, HNLY, HNXH, PO-May, COW-May, ZUW-May, CUW-Aug, ZUW-Aug, COW-Sep, CUW-Sep, and ZUW-Sep

^1^wingless aphids;

^2^winged aphids.

Cotton: *Gossypium* spp.; Hibiscus: *Hibiscus syriacus*; Pomegranate: *Punica granatum*; Chinese prickly ash: *Zanthoxylum bungeanum*; Cucumber: *Cucumis sativus*; Zucchini: *Cucurbita pepo*; Muskmelon: *Cucumis melo*; Kidney bean: *Phaseolus vulgaris*

PN: population number; AP: abbreviation of population; CD: collection data; CL: collection locations; IDN: sample size; MLG: multilocus genotype; A: number of alleles per locus; AR: allelic richness; H_O_^R^: observed heterozygosity calculated with raw data; H_O_^C^: observed heterozygosity calculated with corrected data; H_E_^R^: expected heterozygosity calculated with raw data; H_E_^C^: expected heterozygosity calculated with corrected data; F_IS_: fixation index calculated with raw data; HB: Hebei province; HN: Henan province; SD: Shandong province

### Population genetic structure

**Fig 1:**
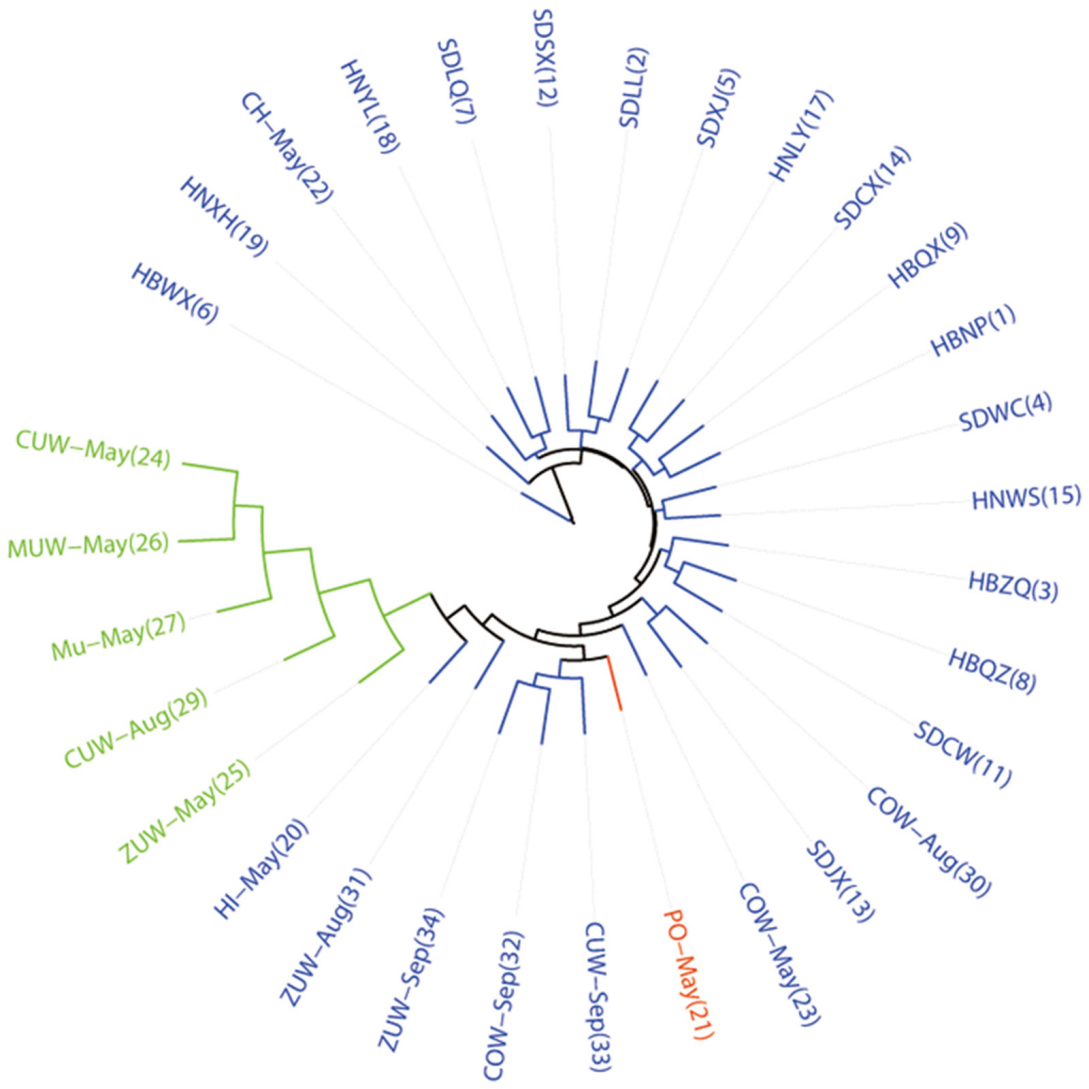
Consensus neighbor-joining tree based on genetic distances. Clades are colored according to the three clusters identified by Structure software when *K* = 3.

There were fewer than five MLGs in populations HBLZ, HNSQ, and KIW-May, which were excluded from population genetic structure analyses. Distinct clusters were not observed in a neighbor-joining tree of 31 *A*. *gossypii* populations based on genetic distances (Fig 1). However, 18 geographic populations appeared to cluster together. Additionally, six melon populations were grouped together, while two other melon populations (i.e., ZUW-Sep and CUW-Sep) and COW-Sep (which were collected in September) were grouped together.

The genetic structure among 18 geographic populations was examined using pairwise comparisons of multilocus F_ST_ with and without ENA correction. The overall F_ST_ value [F_ST_ = 0.103; 95% confidence interval (CI): 0.065–0.145] indicated a moderate level of genetic differentiation among geographic populations (Table 2). Pairwise estimates of F_ST_ calculated between pairs of geographic populations indicated 25.5% of tests for population differentiation were significant (Table 2). Between pairs of geographic populations, 63 (41.2%) pairwise populations were not differentiated (F_ST_ < 0.05), 67 (43.8%) exhibited moderate genetic differentiation (pairwise F_ST_ 0.05 to < 0.15), and 23 (15.0%) revealed considerable genetic differentiation (pairwise F_ST_ 0.15 to < 0.25) (S1 Table). The presence of null alleles only weakly affected pairwise population differentiation in geographic populations (F_ST_^[ENA]^ = 0.101; 95% CI: 0.071–0.141) (Table 2).

**Table 2:** Pairwise F_ST_^[ENA]^ values for all geographic populations (lower-left matrix) and their significance (upper-right matrix).

populations	1	2	3	4	5	6	7	8	9	11	12	13	14	15	17	18	19	32
1		NS	*	NS	NS	NS	*	NS	NS	*	NS	NS	NS	NS	NS	NS	NS	*
2	0.04		NS	NS	NS	NS	NS	NS	NS	*	NS	NS	NS	NS	NS	NS	NS	*
3	0.08	0.08		*	*	*	*	NS	NS	*	NS	NS	NS	NS	NS	NS	NS	*
4	0.07	0.04	0.11		NS	NS	NS	NS	*	*	NS	NS	NS	NS	NS	NS	*	*
5	0.07	0.02	0.14	0.09		NS	NS	NS	NS	*	NS	NS	NS	NS	NS	NS	NS	*
6	0.03	0.06	0.08	0.11	0.10		*	*	NS	*	NS	NS	NS	NS	NS	NS	NS	*
7	0.05	0.08	0.09	0.09	0.10	0.07		*	NS	*	NS	NS	NS	NS	NS	NS	NS	*
8	0.14	0.11	0.13	0.10	0.16	0.16	0.19		NS	*	NS	NS	NS	NS	*	NS	*	*
9	0.01	0.08	0.06	0.12	0.09	0.04	0.03	0.19		NS	NS	NS	NS	NS	NS	NS	NS	*
11	0.12	0.16	0.09	0.18	0.18	0.10	0.13	0.17	0.08		*	NS	NS	*	NS	NS	NS	*
12	0.04	-0.01	0.10	0.06	0.04	0.06	0.07	0.14	0.07	0.16		NS	NS	NS	NS	NS	NS	*
13	0.01	0.03	0.03	0.09	0.12	0.03	0.07	0.17	0.03	0.09	0.04		NS	NS	NS	NS	NS	NS
14	0.01	0.05	0.08	0.12	0.09	0.04	0.05	0.24	0.00	0.11	0.06	0.03		NS	NS	NS	NS	*
15	0.03	0.07	0.08	0.09	0.12	0.03	0.05	0.16	0.03	0.12	0.08	0.01	0.05		NS	NS	NS	*
17	0.00	0.03	0.06	0.07	0.06	0.05	0.03	0.15	0.00	0.09	0.02	0.01	-0.01	0.03		NS	NS	*
18	0.06	0.08	0.09	0.15	0.07	0.04	0.04	0.22	0.03	0.11	0.08	0.04	0.04	0.06	0.03		NS	*
19	0.01	0.04	0.07	0.11	0.07	0.02	0.04	0.18	-0.01	0.09	0.04	0.02	0.00	0.04	0.01	0.02		*
32	0.10	0.09	0.12	0.12	0.18	0.17	0.18	0.19	0.15	0.18	0.15	0.03	0.14	0.14	0.11	0.20	0.13	

*P < 0.05 after sequential Bonferroni correction; NS: non-significant population differentiation; Populations 1–9,11–15, 16–19, and 32 represent the following 20 populations: HBNP, SDLL, HBZQ, SDWC, SDXJ, SDWX, SDLQ, HBQZ, HBQX, HBLZ, SDCW, SDSX, SDJX, SDCX, HNWS, HNSQ, HNLY, HNYL, HNXH, and COW-Sep

The genetic structure among 13 Anyang populations was examined using pairwise comparisons of multilocus F_ST_ with and without the ENA correction. The overall F_ST_ value (F_ST_ = 0.237; 95% CI: 0.187–0.296) indicated moderate genetic differentiation among the Anyang populations. Pairwise F_ST_ estimates between pairs of Anyang populations indicated 50.5% of population differentiation tests were significant (Table 3). Between pairs of Anyang populations, 21 (23.1%) pairwise populations were not differentiated (F_ST_ < 0.05), 21 (23.1%) exhibited moderate genetic differentiation (pairwise F_ST_ 0.05 to < 0.15), 19 (20.9%) revealed considerable genetic differentiation (pairwise F_ST_ 0.15 to < 0.25), and 30 (33.0%) showed extreme genetic differentiation (pairwise F_ST_ > 0.25) (Table 3, S2 Table).

**Table 3:** Pairwise F_ST_^[ENA]^ values for all Anyang populations (lower-left matrix) and their significance (upper-right matrix).

populations	20	21	22	23	24	25	26	27	29	30	31	32	33	34
20		*	*	*	*	*	*	*	*	*	*	*	*	*
21	0.48		*	*	*	*	*	*	*	*	*	*	*	*
22	0.15	0.55		NS	*	*	*	*	NS	NS	*	*	*	*
23	0.08	0.54	0.02		*	NS	NS	*	NS	NS	NS	*	NS	NS
24	0.28	0.72	0.32	0.26		NS	NS	NS	NS	NS	NS	*	*	NS
25	0.11	0.55	0.16	0.07	0.10		NS	NS	NS	NS	NS	*	*	*
26	0.24	0.70	0.29	0.22	0.06	0.06		NS	NS	NS	NS	*	NS	NS
27	0.24	0.70	0.30	0.24	0.11	0.08	0.05		NS	NS	NS	*	*	*
29	0.15	0.65	0.19	0.09	0.04	-0.02	-0.02	0.04		NS	NS	NS	NS	NS
30	0.11	0.56	0.05	0.00	0.37	0.16	0.35	0.36	0.20		NS	NS	NS	NS
31	0.08	0.53	0.08	-0.01	0.24	0.06	0.19	0.23	0.06	0.04		*	NS	NS
32	0.15	0.52	0.14	0.06	0.41	0.22	0.38	0.39	0.27	0.05	0.07		NS	NS
33	0.12	0.50	0.08	0.01	0.32	0.14	0.29	0.31	0.17	0.02	0.01	0.02		NS
34	0.13	0.49	0.11	0.03	0.29	0.12	0.26	0.28	0.15	0.03	0.01	0.04	0.00	

*P < 0.05 after sequential Bonferroni correction; NS, non-significant population differentiation

Populations 20–27 and 29–34 represent the following 14 populations: HI-May, PO-May, CH-May, COW-May, CUW-May, ZUW-May, MUW-May, MU-May, CUW-Aug, COW-Aug, ZUW-Aug, COW-Sep, CUW-Sep, and ZUW-Sep

The presence of null alleles only weakly affected pairwise population differentiation in Anyang populations (F_ST_^[ENA]^ = 0.231; 95% CI: 0.184–0.286) (Table 3).

**Fig 2:**
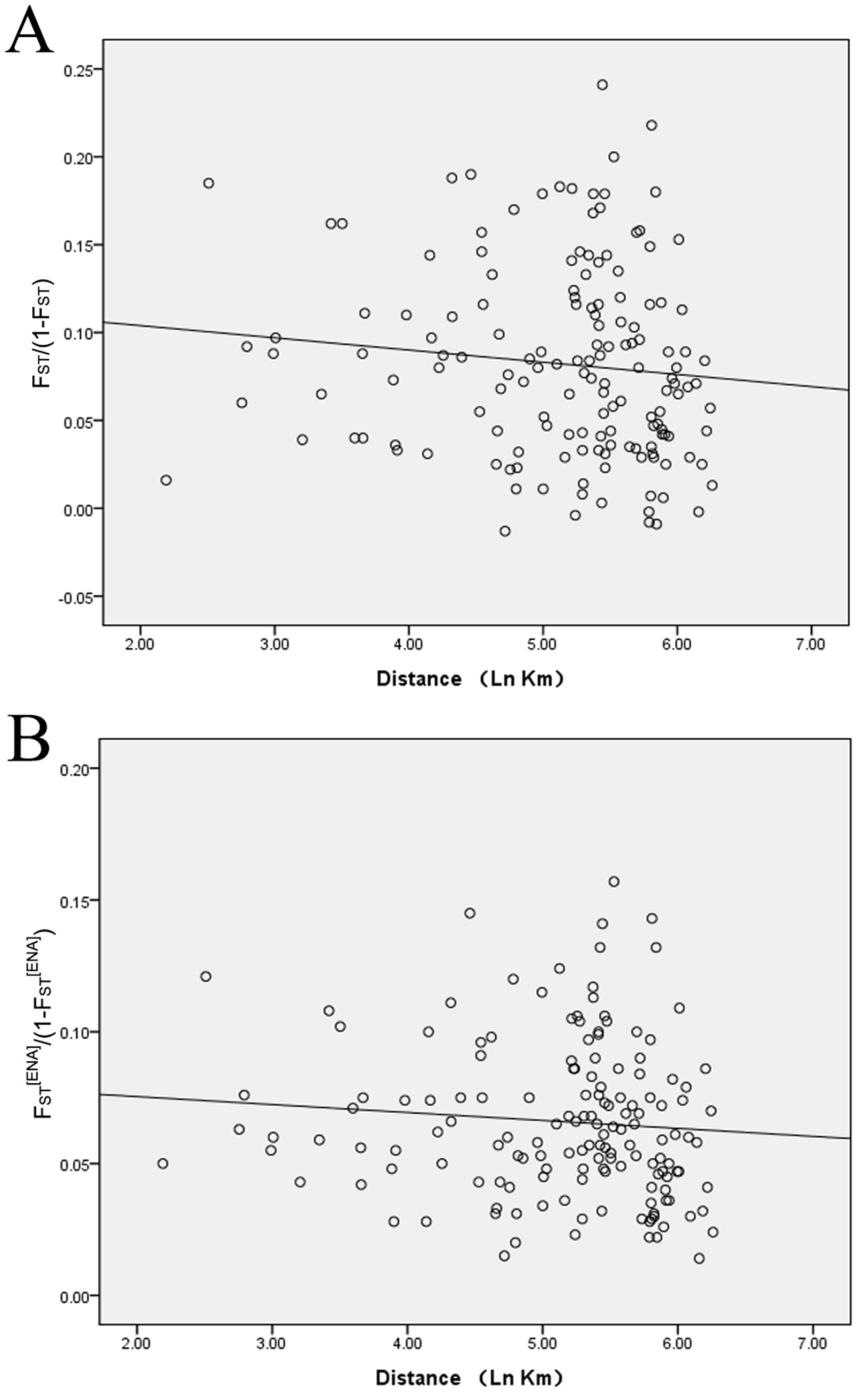
Scatter plots of genetic distance versus geographical distance for pairwise population comparisons. (A) Slatkin’s linearized F_ST_ and the natural logarithm of geographic distance (*r* = −0.086, *P* = 0.291) for all 18 geographic populations. (B) Slatkin’s linearized F_ST_^[ENA]^ and the natural logarithm of geographic distance (*r* = −0.107, *P* = 0.187) for all 18 geographic populations.

The Mantel test of isolation by distance (IBD) revealed there was no significant correlation between Slatkin’s linearized F_ST_ and the natural logarithm of geographic distance (*r* = −0.086, *P* = 0.291) for all 18 geographic populations (Fig 2A). The presence of null alleles only weakly affected the result (*r* = −0.107, *P* = 0.187) (Fig 2B), which indicated the distance between populations was not responsible for the differentiations between populations.

**Fig 3:**
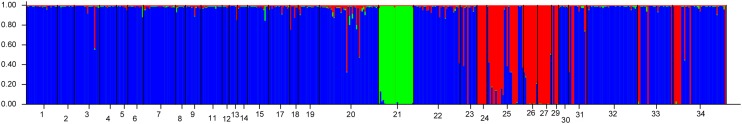
Cluster analysis of *Aphis gossypii* samples from the North China Plain. Assignment of the multilocus genotypes of 31 populations to clusters (K = 3). Each multilocus genotype is represented by a vertical bar. The numbers 1–31 correspond to the following populations: HBNP, SDLL, HBZQ, SDWC, SDXJ, HBWX, SDLQ, HBQZ, HBQX, SDCW, SDSX, SDJX, SDCX, HNWS, HNLY, HNYL, HNXH, HI-May, PO-May, CH-May, COW-May, CUW-May, ZUW-May, MUW-May, MU-May, CUW-Aug, COW-Aug, ZUW-Aug, COW-Sep, CUW-Sep, ZUW-Sep.

Results of the Bayesian analysis of population genetic structures indicated the best dataset partitioning involved three genetic clusters because the modal value of Δ*K* (Evanno method) occurred with *K* = 3 (S1 Fig). The pattern of the three clusters corresponded well with the geographical distributions of the populations and the *A*. *gossypii* aphid colors in the neighbor-joining tree (Fig 1). Cluster analyses of aphid populations were based on the mean proportion of members in a particular cluster. Twenty-five of thirty population proportions were higher than 0.900, with the highest proportion (0.994) observed in cluster 3 of CUW-May. Cluster 1 (mainly blue) included all geographic populations, two primer host populations (HI-May and CH-May), and six Anyang populations (COW-May, COW-Aug, ZUW-Aug, CUW-Sep, COW-Sep, and ZUW-Sep). Cluster 2 (mainly green) comprised one primer host population (PO-May), while cluster 3 (mainly red) included five populations (CUW-May, ZUW-May, MU-May, MUW-May, and CUW-Aug) (Fig 3).

**Fig 4:**
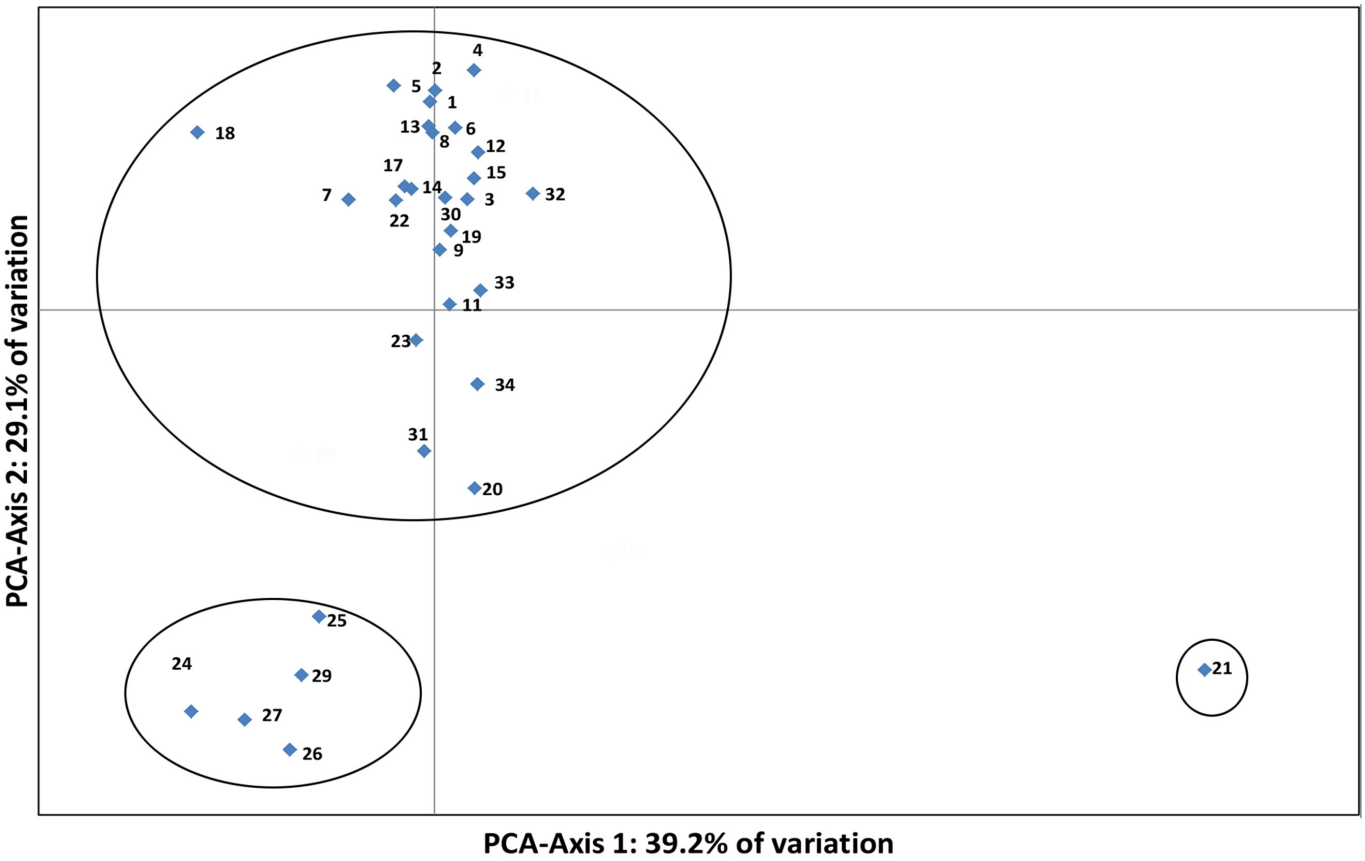
Principal component analysis indicating 31 *Aphis gossypii* populations could be classified into three groups. The numbers 1–31 correspond to the following 31 populations: HBNP, SDLL, HBZQ, SDWC, SDXJ, HBWX, SDLQ, HBQZ, HBQX, SDCW, SDSX, SDJX, SDCX, HNWS, HNLY, HNYL, HNXH, HI-May, PO-May, CH-May, COW-May, CUW-May, ZUW-May, MUW-May, MU-May, CUW-Aug, COW-Aug, ZUW-Aug, COW-Sep, CUW-Sep, ZUW-Sep.

A principal component analysis (PCA) was completed using Nei’s genetic distance pairwise matrix. The PCA results divided the 31 populations into three groups (Fig 4), which were consistent with the cluster analysis results.

**Fig 5:**
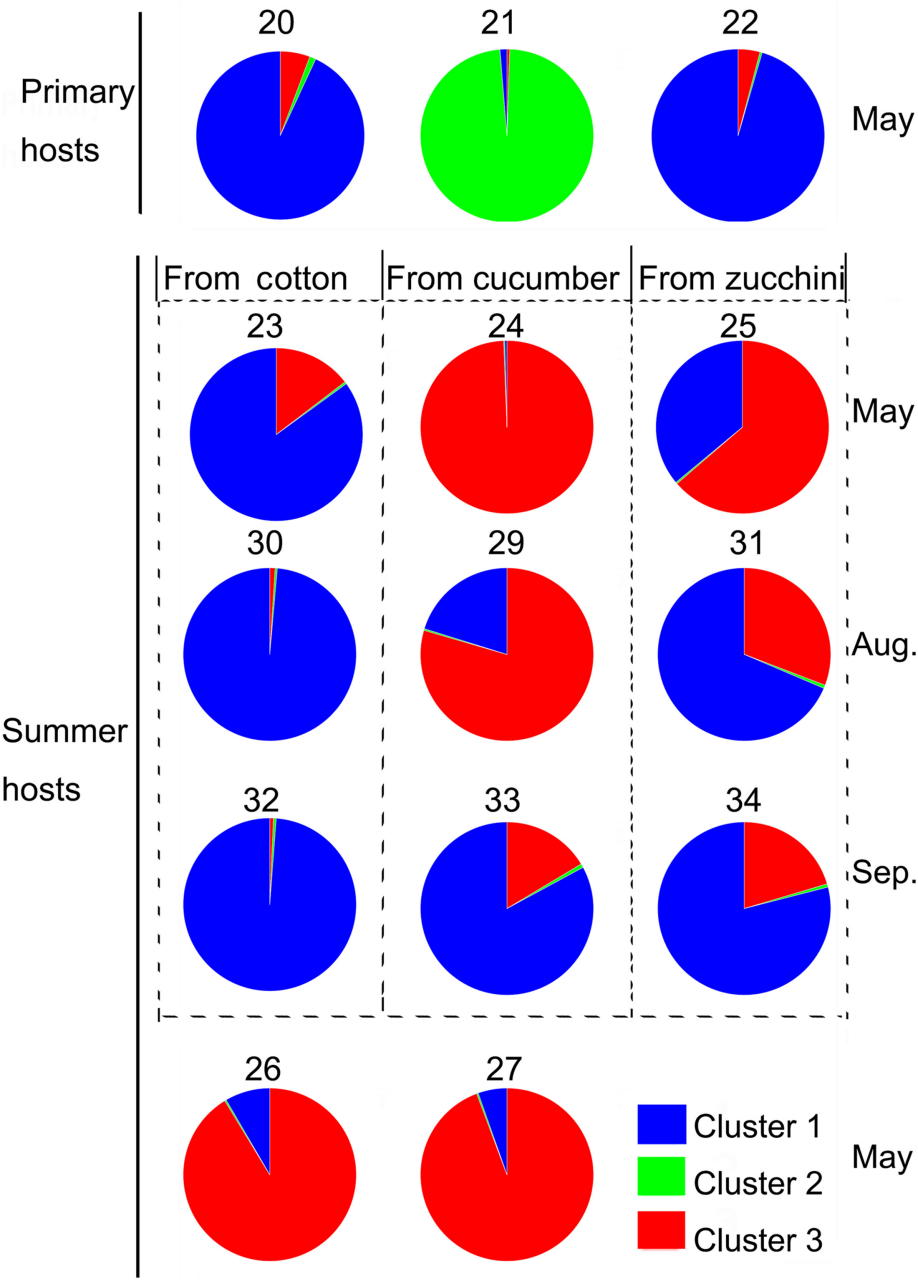
Distribution of *Aphis gossypii* samples according to Bayesian analysis.

The proportions of Anyang populations from three hosts (i.e., cotton, cucumber, and zucchini) depended on the season. In May, the blue proportion of COW-May was 0.850, the red proportion of CUW-May was 0.994, and the blue and red proportions of ZUW-May were 0.360 and 0.636, respectively. In August, the blue proportion of COW-Aug was 0.986, the red proportion of CUW-Aug was 0.795, and the blue and red proportions of ZUW-May were 0.686 and 0.308, respectively. In September, the blue proportion of COW-Sep was 0.988, the red proportion of CUW-Sep was 0.164, the blue proportion of CUW-Sep was 0.829, and the blue and red proportions of ZUW-May were 0.790 and 0.204, respectively (Fig 5).

Analysis of molecular variance (AMOVA) revealed significant differences among the three clusters (*F* = 0.26596, *P* < 0.0001) (Table 4), suggesting there were significant genetic differentiations among the clusters. Differential adaptations to hosts may have led to genetic differentiations. The occurrence of host-based genetic differentiation among Anyang populations was supported by AMOVA (*F* = 0.12975, *P* = 0.0029) (Table 4). Under field conditions, population expansions can be interrupted several times by environmental factors, such as rain and high temperatures, after aphids migrate onto summer hosts. The AMOVA results for comparisons among seasons in Anyang (i.e., May vs. August vs. September) revealed significant differentiations between sampling periods (*F* = 0.04244, *P* = 0.00381) (Table 4).

**Table 4:** Analysis of molecular variance results comparing genetic variations in *Aphis gossypii* populations.

Source of variation	d.f.	Sum of squares	Variance components	Percentage of variation	Fixation indices
(A) Among 3 clusters inferred by STRUCTURE	4	205.941	0.64330Va	26.60	F_CT_ = 0.26596(P<0.0001)
Among populations within groups	31	226.204	0.20024Vb	8.28	F_SC_ = 0.11278(P<0.0001)
Within populations	980	1543.756	1.57526Vc	65.13	F_ST_ = 0.34875(P<0.0001)
(B) Among groups with different season	2	71.147	0.08519Va	4.24	F_CT_ = 0.04244(P = 0.0381)
Among populations within groups	31	360.998	0.34700Vb	17.29	F_SC_ = 0.18052(P<0.0001)
Within populations	980	1543.756	1.57526Vc	78.47	F_ST_ = 0.21529(P<0.0001)
(C) Among groups with different host	7	257.081	0.26538Va	12.97	F_CT_ = 0.12975(P = 0.0029)
Among populations within groups	26	175.063	0.20472Vb	10.01	F_SC_ = 0.11501(P<0.0001)
Within populations	980	1543.756	1.57526 Vc	77.02	F_ST_ = 0.22984(P<0.0001)

^(A)^ Three clusters according to Bayesian analysis

^(B)^ Anyang populations collected in May (HI-May, PO-May, CH-May, COW-May, CUW-May, ZUW-May, MUW-May, MU-May, and KIW-May), August (CUW-Aug, COW-Aug, and ZUW-Aug), and September (COW-Sep, CUW-Sep, and ZUW-Sep)

^(C)^ Anyang populations collected from different hosts, including three primary and five summer hosts

## Discussion

The North China Plain, which is surrounded by mountainous and coastal regions, has long been used to grow cotton. The life cycle of *A*. *gossypii* aphids in this region is considered to be holocyclic [5]. In this study, *A*. *gossypii* samples were collected from cotton plants in late August at 20 locations in the North China Plain. At Anyang, in the middle of the plain, *A*. *gossypii* aphids were sampled from different host species.

Of 31 populations, 16 did not meet the criteria for the HWE, and exhibited significant heterozygote deficiencies. Heterozygosity deficits were also observed for *Sitobion avenae* [9] and *Myzus persicae* [21]. These results suggest the presence of null alleles, along with the parthenogenetic life cycle, resulted in deviations from the HWE.

Our results indicate that the *A*. *gossypii* populations in northern China exhibit greater genotypic diversity in their microsatellite loci than other populations elsewhere in the world [10, 13, 15, 22], except for Japan and Iran [14, 18]. Despite the limited resolution provided by the seven microsatellite loci, we distinguished 507 MLGs among the 906 analyzed individuals. Their M/I ratio of 0.61 was higher than in melon crops at three locations in France and one location in the Lesser Antilles [19], Australia [16], and Iran [18]. A holocyclic life cycle may lead to greater genetic diversity, as observed in aphid populations from southern China and Japan. The species is considered to be anholocyclic in other areas [1, 14]. Similarly, green peach aphid populations are also genetically more diverse in areas where sexual reproduction occurs than in areas where asexual reproduction predominates [23]. Pairwise F_ST_ values indicated there were moderate levels of genetic differentiation among geographic and Anyang populations. Results of the IBD analysis revealed that geographic distance had no effect on *A*. *gossypii* population structure. An AMOVA detected significant differences among the three identified clusters, indicating there was significant genetic differentiation among clusters. *A*. *gossypii* alates migrate to the North China Plain and influence aphid genetic structure. Given the significant genetic differentiation among geographic populations, the migration rate may be low [24], which is also indicated by the Bayesian clustering results.

In this study, the number of MLGs in the three Anyang populations collected in May and August were 245, 27, and 106, with M/I ratios of 0.69, 0.93, and 0.93, respectively. Genetic diversity was strongly influenced by sampling time in populations collected in France [19]. Low clonal diversity may result from heavy selective pressures resulting from repeated treatments of cotton fields with insecticides [13]. Our results indicate M/I ratios at later stages are higher than those of the initial stages in summer hosts. Therefore, weather may have influenced our results because aphids were collected from infested crops in August and September, but heavy summer rains disrupted the aphid reproductive cycle in the North China Plain in 2014. The aphids migrated into the fields in August from both primary and summer hosts. Differential adaptations to hosts may have led to genetic differentiation [19, 22], with M/I ratios of 0.56–1.00 in Anyang populations from various hosts. The M/I ratios were different among geographic populations from cotton, perhaps owing to different selective pressures, such as insecticide treatments [13]. In three Anyang aphid populations from cotton that were not exposed to pesticides, the M/I ratios were above 0.92, which was higher than the ratios of most populations collected from cotton fields.

Many studies have reported the existence of host-specific *A*. *gossypii* biotypes. This specialization has occurred worldwide on the same crops. A microsatellite study of *A*. *gossypii* in northern Cameroon revealed that cotton and cucurbits were colonized by distinct groups of clonal genotypes [10]. A similar study in Tunisia determined that cultivated crops of the families Cucurbitaceae and Solanaceae were infested by specific *A*. *gossypii* genotypes [22]. Additionally, Carletto et al. [13] identified five host races using eight microsatellites. Previous studies classified *A*. *gossypii* aphids in southern China in two groups based on whether they colonized cotton or cucumber [25]. In this study, a Bayesian analysis of population genetic structures revealed three genetic clusters. This clustering was confirmed using PCA results, which divided the 31 populations into three groups consisting of the same populations as the genetic clusters. Our findings indicate the 31 populations could be classified into one of three biotypes according to host species (i.e., cotton, cucumber, and pomegranate biotypes). Many studies have described host-specific *A*. *gossypii* biotypes, but a lack of standardization has prevented comparisons of biotypes [7]. Microsatellite analyses may lead to a standardization protocol that enables comparisons among biotypes.

In China, there are five summer hosts for *A*. *gossypii*, while hibiscus, pomegranate, and Chinese prickly ash serve as the main primary hosts. A previous study concluded *A*. *gossypii* from hibiscus plants preferred cotton over cucumber in southern China [25]. Bayesian analysis results revealed that populations from hibiscus and Chinese prickly ash plants were mainly blue, indicating these plants are the primary hosts of the cotton biotype of *A*. *gossypii*. Meanwhile, the red proportion of aphid populations from hibiscus and Chinese prickly ash plants was about 0.050, suggesting these plants are also the primary hosts of the cucumber biotype. The aphid populations from pomegranates were mainly green, and no summer hosts were identified.

Because of broad phenotypic plasticity, aphids can be opportunistic and colonize different plant hosts [12], with differential adaptations to hosts resulting in genetic differentiation. In Anyang populations, clonal diversity depended on the plant hosts [18]. Because *A*. *gossypii* completes a holocyclic life cycle in Anyang, the seasons were another factor affecting genetic diversity. Results of the AMOVA between seasons revealed significant differences between sampling periods. Bayesian analysis also detected seasonal effects. For the three summer hosts (i.e., cotton, cucumber, and zucchini), the seasons obviously affected population genetic structures and host biotypes. The aphid populations may not have undergone host plant-dependent selective filtering, which may have been responsible for the differences between alate and apterous populations [19].

In conclusion, the *A*. *gossypii* populations in northern China exhibit considerable genotypic diversity. Moderate levels of genetic differentiation among geographic populations and host plants, and geographic distances did not affect *A*. *gossypii* population structures. Our findings indicate the 31 populations belonged to three host biotypes (i.e., cotton, cucumber, and pomegranate biotypes). Additionally, the seasons clearly influenced the population genetic structure diversity in Anyang aphids.

## Materials and Methods

### Ethics Statement

The cotton-melon aphid is an insect pest of many plant species. Because studies of *A*. *gossypii* may provide a new method to control this pest, such investigations are welcomed by farmers. No specific permits were required for field studies, which did not involve endangered or protected species.

### Sample collection and DNA extraction

Samples of wingless *A*. *gossypii* aphids were collected from cotton fields in northern China in late August 2014. A total of 564 individuals were sampled at 20 sites, including six populations in Hebei province, sampling locations were Nanpi (HBNP; 37°58'53.1N, 116°49'34.1E), Zhaoqiang (HBZQ; 37°31'25.4N, 115°39'22.5E), Weixian (HBWX; 36°58'59.6N, 115°17'37.7E), Quzhou (HBQZ; 36°47'02.6N, 115°00'06.3E), Qiuxian (HBQX; 36°47'02.6N, 115°00'06.3E), Linzhang (HBLZ; 36°20'11.3N, 114°33'47.4E); six in Henan province, sampling locations were Weishi (HNWS; 34°03'42.6N, 114°27'10.9E), Shangqiu (HNSQ; 34°31'54.9N, 115°42'26.8E), Luyi (HNLY; 33°50'33.1N, 115°32'41.1E), Yanling (HNYL; 33°50'30.5N, 114°14'11.4E), Xihua (HNXH; 33°45'58.6N, 114°14'14.1E) (including COW-Sep, which was collected in September in Anyang (36°5'34.8"N, 114°31'47.19"E)); and eight in Shandong province, sampling locations were Laoling (SDLL; 37°37'18.8N, 117°09'13.5E), Wucheng (SDWC; 37°11'35.2N, 116°05'42.1E), Xiajing (SDXJ; 37°00'43.2N, 116°00'56.3E), Linqing (SDLQ; 36°53'51.5N, 115°49'54.4E), Chengwu (SDCW; 35°04'23.7N, 116°07'49.7E), Shanxian (SDSX; 34°47'58.7N, 115°59'57.6E), Jingxiang (SDJX; 35°01'18.7N, 116°18'22.5E), Caoxian (SDCX; 34°48'21.6N, 115°49'27.6E). Only one individual per plant from 8–48 cotton plants per site was collected to avoid sampling the offspring of a single female.

Populations were collected during different seasons and from various hosts in Anyang (36°5'34.8"N, 114°31'47.19"E). Three wingless aphid populations from the primary hosts, hibiscus (HI-May), pomegranate (PO-May), and Chinese prickly ash (CH-May), were collected in May 2014 before they switched to the summer hosts. Five summer hosts (i.e., cucumber, zucchini, muskmelon, kidney bean, and cotton) were planted in April 2014 on a farm where no pesticides were used (36°5'34.8"N, 114°31'47.19"E). Five adult winged aphid populations were collected in May as soon as they appeared on the following summer host plants: cucumber (CUW-May), zucchini (ZUW-May), muskmelon (MUW-May), kidney bean (KIW-May), and cotton (COW-May). One wingless aphid population was collected from muskmelon (MU-May). In late August and September, six winged aphid populations were collected from cotton (COW-Aug and COW-Sep), cucumber (CUW-Aug and CUW-Sep), and zucchini (ZUW-Aug and ZUW-Sep). Only one aphid per plant was sampled (Table 1).

Samples were placed in 95% ethanol and stored at room temperature. Total DNA from individual aphids was extracted using the TIANamp Genomic DNA Kit (Tiangen, Beijing, China) following the manufacturer’s protocol. The purified DNA samples were stored at −20°C. Because *A*. *gossypii* and congeneric species are not easily distinguished morphologically, sample identities were confirmed by PCR and sequencing prior to genotyping [20].

### Microsatellite genotyping

Each aphid was genotyped at eight microsatellite loci (i.e., Ago24-FAM, Ago53-HEX, Ago59- TAMRA, Ago66-HEX, Ago69-TAMRA, Ago84-ROX, Ago89-ROX, and Ago126-FAM) using primers designed for *A*. *gossypii* [15]. The first PCR using primers specific for seven loci (i.e., Ago53, Ago59, Ago66, Ago69, Ago84, Ago89, and Ago126) was completed in a final volume of 20 μL containing 10 μL 2× Taq Premix (TaKaRa, Dalian, China), 0.2 μM each primer, 1 μL DNA, and water. A second PCR using primers specific for Ago24 was completed using the same conditions, except for a lower primer concentration (0.1 μM). Amplifications were conducted in a Mastercycler (Eppendorf, Hamburg, Germany) as previously described [15]. Allele resolution and analyses were completed using an ABI 3730 automated sequencer with GeneScan-500 ROX (Applied Biosystems, Foster City, CA, USA) as the internal size standard. Results were interpreted using GeneScan v. 3.2 (Applied Biosystems).

### Data analyses

Genotypes at seven loci (i.e., Ago24, Ago53, Ago59, Ago66, Ago69, Ago89, and Ago126) for each aphid from each population formed a different MLG according to GenClone v. 2.0 software [26]. We used GenePop v. 4.3 [27] to analyze deviations from the HWE by considering only one sample per MLG (i.e., clones were excluded) [28]. The data were formatted for subsequent statistical analysis using CONVERT v. 1.31 software [29]. To examine the possible occurrence of null alleles at microsatellite loci, the van Oosterhout algorithm of Micro-Checker v. 2.2.3 was used [30]. Descriptive statistics, including the number of alleles per locus, allelic richness, and fixation index (F_IS_), were estimated using FSTAT v. 2.9.3.2 software. The F_IS_ value ranges from −1 to +1, with negative F_IS_ values indicating heterozygote excess (outbreeding) and positive values indicating heterozygote deficiency (inbreeding) relative to HWE expectations [31]. The H_E_ and H_O_ values were determined with GenALEX v. 6, which was also used to complete the PCA [32]. To minimize any biases in the genetic diversity statistics induced by null alleles, the corrected dataset was used to determine the H_O_, H_E_, and F_IS_ statistics. Additionally, the HWE test involved the EM and INA methods [33].

A neighbor-joining tree using genetic distance was constructed with PHYLIP v. 3.696 (University of Washington; http://evolution.genetics.washington.edu/phylip.html) and 5,000 bootstraps to estimate the phylogenetic relationships among populations. An F_ST_ value of zero indicates a lack of divergence between populations, while an F_ST_ of one indicates complete isolation of populations. Therefore, pairwise F_ST_ values for each comparison of populations were calculated using FSTAT. The ENA method was also used to obtain unbiased pairwise F_ST_ values (F_ST_^[ENA]^) using the FreeNA program [33]. A Mantel test of IBD was completed in GenePop, with significance tests performed with 1,000 permutations.

Population structures were analyzed using the Bayesian clustering algorithm of Structure v. 2.3.4 software [34]. The Markov chain Monte Carlo simulation was run 20 times for each *K* value (1 ≤ *K* ≤ 10) for 500,000 iterations after a burn-in period of 750,000. The optimal *K* value was determined using the highest Δ*K* value as described previously [35]. The Δ*K* value was calculated using Structure Harvester v. 0.56.3 [36]. Population genetic variances were investigated further by AMOVA [37] using Arlequin v. 3.5.1.3 [38].

## Supporting Information

S1 Fig Scatter plots of ΔK. (TIF)

S1 Table Pairwise F_ST_ values for all *Aphis gossypii* geographic populations. (XLSX)

S2 Table Pairwise F_ST_ values for all *Aphis gossypii* Anyang populations. (XLSX)
