# Supplementary figures and images for: The Distribution and Host Shifts of Cotton-Melon Aphids in Northern China
Source: PLoS One. 2016 Mar 22;11(3):e0152103. doi: 10.1371/journal.pone.0152103 (PMC4803350; doi:10.1371/journal.pone.0152103)

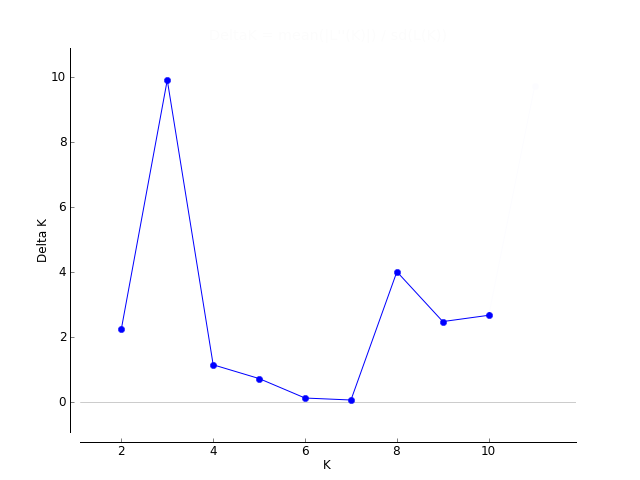

Supplement: S1 Fig — (TIF) [file pone.0152103.s001.tif]
